# Individual classification of ADHD children by right prefrontal hemodynamic responses during a go/no-go task as assessed by fNIRS

**DOI:** 10.1016/j.nicl.2015.06.011

**Published:** 2015-07-09

**Authors:** Yukifumi Monden, Ippeita Dan, Masako Nagashima, Haruka Dan, Minako Uga, Takahiro Ikeda, Daisuke Tsuzuki, Yasushi Kyutoku, Yuji Gunji, Daisuke Hirano, Takamichi Taniguchi, Hideo Shimoizumi, Eiju Watanabe, Takanori Yamagata

**Affiliations:** a Department of Pediatrics, Jichi Medical University, 3311-1 Yakushiji, Shimotsuke, Tochigi 329-0498, Japan; b Functional Brain Science Laboratory, Jichi Medical University, 3311-1 Yakushiji, Shimotsuke, Tochigi 329-0498, Japan; c Department of Neurosurgery, Jichi Medical University, 3311-1 Yakushiji, Shimotsuke, Tochigi 329-0498, Japan; d Applied Cognitive Neuroscience Laboratory, Chuo University, 1-13-27 Kasuga, Bunkyo, Tokyo 112-8551, Japan; e Department of Pediatrics, International University of Health and Welfare, 537-3 Iguchi, Nasushiobara, Tochigi 329-2763, Japan; f International University of Health and Welfare, Otawara, Tochigi, Japan; g Rehabilitation Center, International University of Health and Welfare, 2600-1 Kitakanemaru, Otawara, Tochigi 324-8501, Japan

**Keywords:** Developmental syndromes, Optical topography, Response inhibition, Endophenotype, Discrimination analysis

## Abstract

While a growing body of neurocognitive research has explored the neural substrates associated with attention deficit hyperactive disorder (ADHD), an objective biomarker for diagnosis has not been established. The advent of functional near-infrared spectroscopy (fNIRS), which is a noninvasive and unrestrictive method of functional neuroimaging, raised the possibility of introducing functional neuroimaging diagnosis in young ADHD children. Previously, our fNIRS-based measurements successfully visualized the hypoactivation pattern in the right prefrontal cortex during a go/no-go task in ADHD children compared with typically developing control children at a group level. The current study aimed to explore a method of individual differentiation between ADHD and typically developing control children using multichannel fNIRS, emphasizing how spatial distribution and amplitude of hemodynamic response are associated with inhibition-related right prefrontal dysfunction. Thirty ADHD and thirty typically developing control children underwent a go/no-go task, and their cortical hemodynamics were assessed using fNIRS. We explored specific regions of interest (ROIs) and cut-off amplitudes for cortical activation to distinguish ADHD children from control children. The ROI located on the border of inferior and middle frontal gyri yielded the most accurate discrimination. Furthermore, we adapted well-formed formulae for the constituent channels of the optimized ROI, leading to improved classification accuracy with an area under the curve value of 85% and with 90% sensitivity. Thus, the right prefrontal hypoactivation assessed by fNIRS would serve as a potentially effective biomarker for classifying ADHD children at the individual level.

## Highlights

• Objective neuro-functional biomarker to diagnose ADHD has not been established.
• We measured right prefrontal fNIRS signals with a go/no-go task execution executed.
• We assessed the accuracy of classification between ADHD and healthy control.
• We found the way of classification with 90% sensitivity of diagnostic prediction.
• Our results would provide screening tool clinically applicable for ADHD children.

## 1 Introduction

Attention deficit hyperactivity disorder (ADHD) is one of the most commonly diagnosed neurodevelopmental disorders in children. ADHD affects between 3 and 12% of elementary school children with behavioral symptoms being characterized by hyperactivity, impulsivity and inattention (Dittmann et al., 2009; Thomas et al., 2015). ADHD symptoms are often identified between the ages of 4 and 6 (Drechsler et al., 2005). ADHD children tend to suffer from emotional problems, which can lead to academic difficulties and antisocial behaviors (Classi et al., 2012). Moreover, such ADHD symptoms become chronic in 75–85% of patients (Lahey et al., 2004). Consequently, ADHD often persists into adolescence and adulthood, presenting patients with difficulty in educational and vocational performance and increased risk of depression and suicide (Chronis-Tuscano et al., 2010). Indeed, 4–5% of adults have recently been reported to have ADHD (Safren et al., 2010). Therefore, early diagnosis with appropriate treatment is considered important to improve the quality of life of ADHD patients (Power et al., 2003). Current ADHD diagnosis mainly depends on interview-based evaluation of the degrees of the phenotypes listed in the diagnostic criteria of the DSM-IV and DSM-5 as observed by a patient's parents or teachers (Wehmeier et al., 2010; Zhu et al., 2008). However, the interview-based assessment often entails subjective evaluation, which has a risk of underdiagnosis and overdiagnosis of ADHD symptoms (Batstra et al., 2012; Bruchmuller et al., 2012). Because of the technical limitations of relying on clinical interviews based on clinical observation, as well as parents and teachers ratings in ADHD diagnosis, the identification of a biological marker for early and objective diagnosis (Dillo et al., 2010) has been eagerly awaited (Wehmeier et al., 2010; Wolraich et al., 2013; Zhu et al., 2008).

One potential approach is the use of noninvasive functional neuroimaging modalities, such as functional magnetic resonance imaging (fMRI), SPECT, PET, and, most recently, functional near-infrared spectroscopy (fNIRS) to present candidate biomarkers for ADHD. Previous fMRI studies have elucidated neurological dysfunction of frontostriatal network in ADHD patients (Bush et al., 1999; Dillo et al., 2010; Durston et al., 2003; Rubia et al., 1999; Vaidya et al., 1998). In addition, a whole-brain tractography analysis reported that frontal, striatal and cerebellar regions are related with core neurological dysfunction in ADHD (Hong et al., 2014).

Among modalities, fNIRS provides robust advantages such as its compactness (useful in confined experimental settings), affordable price, tolerance to body motion and accessibility (Herrmann et al., 2004; Herrmann et al., 2005; Hock et al., 1997; Matsuo et al., 2000; Matsuo et al., 2003; Moriai-Izawa et al., 2012; Okamoto et al., 2004b; Okamoto et al., 2006; Shinba et al., 2004; Strangman et al., 2002a; Suto et al., 2004), and thus is considered suitable for the clinical assessment of ADHD children.

In a series of studies making the most of fNIRS's merits, we have explored the neural substrate of inhibitory and attentional controls in school-aged ADHD children. Importantly, the cumulative post-scan exclusion rate of our previous fNIRS studies (all studies) is less than 5% of a total of 81 right-handed ADHD children and 69 control subjects including 6 years old (Monden et al., 2012a; Monden et al., 2012c; Nagashima et al., 2014a; Nagashima et al., 2014b; Nagashima et al., 2014c). This is far lower than the rejection rate of fMRI studies, which is typically 50% for ADHD subjects 6 years old and older and 30% for the corresponding normal controls. These high rejection rates are mainly due to motion and lack of compliance.

We monitored the oxy-hemoglobin signal changes of ADHD children (6–14 years old) performing go/no-go or oddball tasks to examine inhibitory and attentional controls, respectively. We also included non-medicated, age- and gender-matched normal controls. In the control subjects, the go/no-go task recruited the right inferior and middle prefrontal gyri (IFG/MFG), but this activation was absent in pre-medicated ADHD children. The reduction of right IFG/MFG activation was acutely normalized after administration of MPH (OROS-methylphenidate, commercially available as Concerta) or ATX (atomoxetine, commercially available as Strattera) (Monden et al., 2012c; Nagashima et al., 2014b). Regarding the oddball task, in addition to the right IFG/MFG, the right inferior parietal cortices were recruited in the control subjects. However, these activations were absent in pre-medicated ADHD children. While ATX normalized both right prefrontal and parietal activations in ADHD children upon medication, MPH normalization was confined to the right IFG/MFG (Nagashima et al., 2014a; Nagashima et al., 2014c).

From a genetic perspective, genetic factors also have been reported to play an important role in the development and course of ADHD disorder. In recent years considerable studies on different candidate genes for ADHD have been identified such as the catechol-O-methyltransferase (COMT) gene (Tomlinson et al., 2015), the dopamine active transporter 1 gene (DAT1, also known as SLC6A3) and the dopamine receptor D4 (DRD4) gene (Faraone et al., 2014). These genes are thought to involved in monoamine system, and their dysfunction in the prefrontal cortex is considered to be the core-pathomechanism of ADHD.

Thus, fNIRS successfully visualized differential neural substrates for ADHD and typically developing control children in inhibitory and attentional controls in group analyses. While both go/no-go and oddball tasks recruited the right IFG/MFG, the activation was typically greater for the go/no-go task (Cohens d: 1.16, go/no-go task; 0.98, oddball task) (Monden et al., 2012c; Nagashima et al., 2014a; Nagashima et al., 2014b; Nagashima et al., 2014c). These findings led us to postulate that right IFG and MFG activations as observed using fNIRS, especially for a go/no-go task, might be used as an objective neuro-functional biomarker to diagnose school-aged ADHD children possibly at the individual level. Therefore, our next undertaking is to quantify the inhibition-related dysfunction in ADHD children at an individual level.

In order to realize a highly accurate classification between ADHD and typically developing control children at an individual level, we have to deal with two issues. First, while go/no-go task-related activation in control subjects is conspicuously great at the single channel located between the right MFG and the IFG in group analyses, activation patterns vary across individuals. Such individual spatial variability necessitates that we determine which areas of the prefrontal cortices should be assessed to comprise the regions of interest (ROIs) to represent areas of inhibition-related cortical dysfunction in ADHD children. Second, although go/no-go task-related activation in the control subjects has a large effect size with group analyses, amplitude of activation varies across individuals. Thus, a cut-off amplitude for cortical activation needs to be identified for each ROI mentioned above in order to differentiate ADHD children from control children. Thus, making the best use of multichannel measurements, we adapted well-formed formulae to analyze the constituent CHs of the optimized ROIs, and assessed whether a specific logic could improve the efficacy of classification.

Hence, in this study, we explore a method for individually classifying ADHD children in comparison to typically developing children by using fNIRS, emphasizing how spatial distribution and amplitude of hemodynamic response associated with go/no-go task execution can be utilized. In so doing, we explore the feasibility of providing objective diagnostic tools that are clinically applicable for school-aged ADHD children.

## 2 Materials and methods

### 2.1 Subjects

Thirty clinically referred, right-handed Japanese children with a mean age of 9.1 (SD 2.6, range 6–15 years; gender: 25 male and 5 female) who met the DSM-IV criteria for pre-medicated ADHD participated in the study (Table 1). Some subjects had also been included in our previous studies (Monden et al., 2012a; Monden et al., 2012b). The Wechsler Intelligence Scale of Children – Third Edition (WISC-III) full IQ scores of subjects were all over 70 (mean 95.5, SD 13.0, range 75–126). Thirty right-handed typically developing control subjects were matched with the pre-medicated ADHD subjects according to age (mean 9.7, SD 2.3, range 6–14 years) and gender (20 males and 10 females). IQs of controls (mean 109.3, SD 14.9, range 82–150) were significantly (*t* = 3.802, *p* < 0.01) higher than those of pre-medicated ADHD subjects. All children and their parents gave oral consent for their participation in the study. Written consent was obtained from the guardians of all subjects according to the latest version of the Declaration of Helsinki. The study was approved by the Ethics Committees of Jichi Medical University Hospital, and the International University of Health and Welfare.

### 2.2 Experimental design

**Fig. 1:**
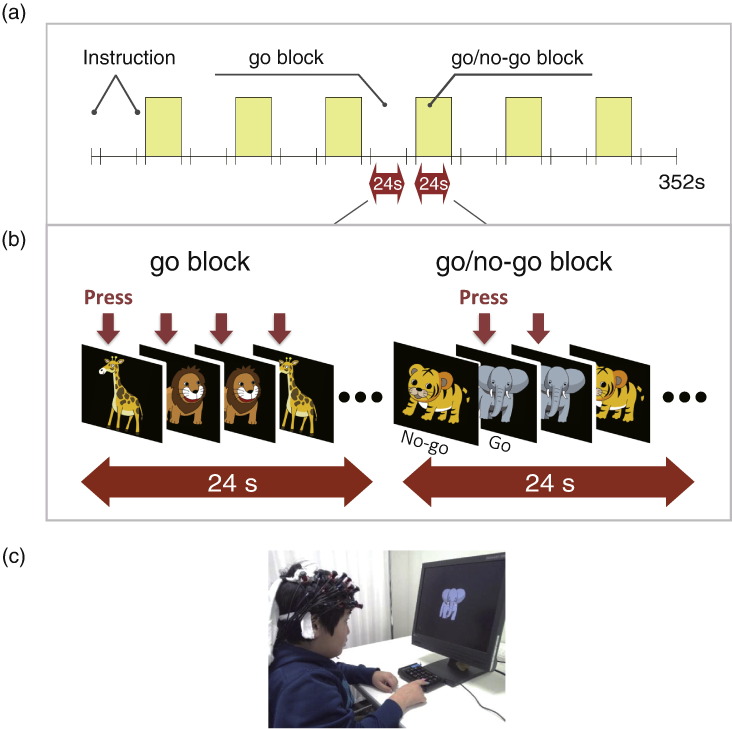
Summary of experimental procedure. Brain activity was measured while ADHD and control subjects performed a go/no-go task.

We examined inhibition-related hemodynamic cortical activation while the subjects performed a go/no-go task. We examined pre-medicated ADHD and control subjects once without medication; all pre-medicated ADHD subjects underwent a washout period of more than 2 days for MPH or ATX. The procedure consisted of 6 block sets, containing alternating go (baseline) and go/no-go (target) blocks. Each block lasted 24 s and was preceded by instructions displayed for 3 s, giving an overall block-set time of 54 s and a total session time of about 6 min. In the go block, we presented subjects with a random sequence of two pictures and asked them to press a button for both pictures. In the go/no-go block, we presented subjects with a no-go picture 50% of the time, thus requiring subjects to respond to half the trials (go trials) and inhibit their response to the other half (no-go trials). Specifically, the instructions read in Japanese, “You should press the button as quickly as you can. Remember you want to be quick but also accurate, so do not go too fast.” Participants responded using the forefinger of their right hand. A go/no–go ratio of 50% was selected as it has been most often used in previous neuroimaging studies (Dillo et al., 2010; Herrmann et al., 2005; Liddle et al., 2001; Menon et al., 2001; Vaidya et al., 1998). We presented pictures sequentially for 800 ms with an inter-stimulus interval of 200 ms during go and go/no-go blocks. At the beginning of each block, we displayed instructions (e.g., “press for giraffe or lion” for go conditions and “do not press for tiger” for go/no-go conditions) for 3 s to inform the subject about the new block. Each subject performed a practice block before any measurements to ensure their understanding of the instructions (Fig. 1). The Experimental design was as previously described (Monden et al., 2012a; Monden et al., 2012c).

### 2.3 fNIRS Measurements

We used the multichannel fNIRS system ETG-4000 (Hitachi Medical Corporation, Kashiwa, Japan), utilizing two wavelengths of near-infrared light (695 and 830 nm). We analyzed the optical data based on the modified Beer–Lambert Law (Cope et al., 1988) as previously described (Maki et al., 1995). This method enabled us to calculate signals reflecting the oxygenated hemoglobin (oxy-Hb), deoxygenated hemoglobin (deoxy-Hb), and total hemoglobin (total-Hb) signal changes, obtained in units of millimolar·millimeter (mM mm) (Maki et al., 1995).

For statistical analyses, we focused on the oxy-Hb signal because of its higher sensitivity to changes in cerebral blood flow than that of deoxy-Hb and total-Hb signals (Hoshi, 2003; Hoshi et al., 2001; Strangman et al., 2002b), its higher signal-to-noise ratio (Strangman et al., 2002b), and its higher retest reliability (Plichta et al., 2006).

**Fig. 2:**
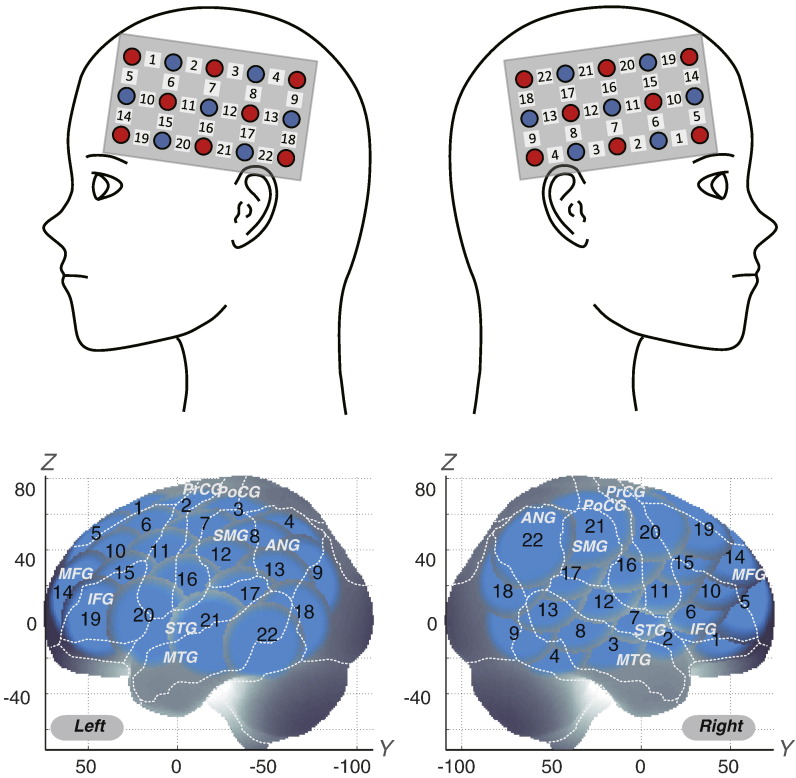
Spatial profiles of fNIRS channels. Left (a) and right (b) side views of the probe arrangements are exhibited with fNIRS channel orientation. Detectors are indicated with blue circles, illuminators with red circles, and channels with white squares. Corresponding channel numbers are shown in black. Channel locations on the brain are exhibited for both left and right side views. Probabilistically estimated fNIRS channel locations (centers of blue circles) for control and ADHD subjects, and their spatial variability (SD, radii of the blue circles) associated with the estimation are depicted in MNI space.

We set the fNIRS probes so that they covered the lateral prefrontal cortices and inferior parietal lobe, referring to previous studies (Garavan et al., 1999; Herrmann et al., 2004; Herrmann et al., 2005; Liddle et al., 2001; Rubia et al., 2003). Specifically, we used two sets of 3 × 5 multichannel probe holders that consisted of eight illuminating and seven detecting probes arranged alternately at an inter-probe distance of 3 cm. This resulted in 22 channels (CH) per set. We defined the midpoint of a pair of illuminating and detecting probes as a channel location. We attached the bilateral probe holders in the following manner: (1) their upper anterior corners, where the left and right probe holders were connected by a belt, were symmetrically placed across the sagittal midline; (2) the lower anterior corners of the probe holder were placed over the supraorbital prominence; and (3) the lower edges of the probe holders were attached at the upper part of the auricles (Fig. 2). For spatial profiling of fNIRS data, we adopted virtual registration (Tsuzuki and Dan, 2014; Tsuzuki et al., 2007) for registering fNIRS data to MNI standard brain space (Brett et al., 2002). Briefly, this method enables us to place a virtual probe holder on the scalp based on a simulation of the holder's deformation and the registration of probes and channels onto reference brains in an MRI database (Okamoto et al., 2004a; Okamoto and Dan, 2005). Specifically, we measured the positions of channels and reference points, consisting of the Nz (nasion), Cz (midline central) and left and right preauricular points, with a 3D-digitizer in real-world (RW) space. We affine-transformed the RW reference points to the corresponding reference points in each entry in reference to the MRI database in MNI space. Adopting these same transformation parameters allowed us to obtain the MNI coordinates for the fNIRS channels and the most likely estimate of the locations of given channels for the group of subjects together with the spatial variability associated with the estimation (Singh and Dan, 2006). Finally, we estimated macroanatomical labels using a Matlab function that reads labeling information coded in a macroanatomical brain atlas, LBPA40 (Shattuck et al., 2008) and Brodmann's atlas (Rorden and Brett, 2000).

### 2.4 Analysis of fNIRS data

We preprocessed individual timeline data for the oxy-Hb signals of each channel with a first-degree polynominal fitting and high-pass filter using cut-off frequencies of 0.01 Hz to remove baseline drift, and a 0.8 Hz low-pass filter to remove heartbeat pulsations. Note that Hb signals analyzed in the current study do not directly represent cortical Hb concentration changes, but contain an unknown optical path length that cannot be measured. Direct comparison of Hb signals among different channels and regions should be avoided as optical path length is known to vary among cortical regions (Katagiri et al., 2010). From the preprocessed time series data, we computed channel-wise and subject-wise contrasts by calculating the inter-trial mean of differences between the oxy-Hb signals for target (4–24 s after go/no-go block onset) and baseline (14–24 s after go block onset) periods. For the six go/no-go blocks, we visually inspected the motions of the subjects and removed the blocks with sudden, obvious, discontinuous noise. Furthermore, we excluded the data with the subject's having the removed blocks more than 3 of 6 blocks.

### 2.5 Individual fNIRS-based classification

Based on the individual and channel-wise oxy-Hb signal contrast measured using fNIRS, a method for quantitative analysis was explored so as to enable a higher disease classification rate between ADHD and typically developing children. Because statistically decreased cortical activation was consistently present in 1) the right side and 2) the prefrontal area in ADHD children in our previous studies (Monden et al., 2012a; Monden et al., 2012c; Nagashima et al., 2014b), we adopted these areas as ROIs in order to explore a robust classification parameter.

We set the following four anatomical ROIs: Region 1 (CH 1 to 22) covered all measured channels in the right hemisphere. Region 2 (CHs 1, 2, 5, 6, 7, 10, 11, 14, 15, 16, 19 and 20) included all the channels that were probabilistically located in the right prefrontal cortex either entirely or partly. Regions 3 and 4 were determined in exploratory ways; their specific constituents will be described in the Results section. Region 3 consisted of the CHs that were significantly activated during go/no-go task execution in the group analysis of normal control subjects. Specifically, we selected the experimental ROIs where significant oxy-Hb difference was observed between ADHD and control subjects in group comparison.

Then, to screen the channels involved in go/no-go tasks in normal control and ADHD subjects, we performed paired *t*-tests (two-tails) on target vs. baseline contrasts. We set the statistical threshold at 0.05 with Bonferroni correction for family-wise errors. Region 4 consisted of the CHs that exhibited significantly larger go/no-go-task-associated activation in the normal control children than in ADHD children. Among the channels that constitute Region 4, we performed comparisons between control and ADHD children. We performed independent two-sample t-tests (two-tails) on these contrasts with a statistical threshold of *p* < 0.05. For each CH, we assessed the integral value of oxy-Hb signal. This value represents the size of the hemodynamic response during the inter-trial mean of differences between the oxy-Hb signals (4–24 s after go/no-go block onset) and baseline (14–24 s after go block onset) periods in each CH. We averaged each CH's integral value for the four ROIs presented above.

Next, we explored setting a cut-off value to distinguish ADHD children from control children with higher accuracy. For this purpose, a conservative receiver operating characteristic (ROC) analysis was performed and used to generate simple indices of fNIRS-based oxy-Hb signal patterns for individual ADHD diagnosis.

Specifically, it was determined whether 30 ADHD and 30 normal control subjects were below or above a given cut-off value for oxy-Hb signal (detected ADHD or detected normal). The subjects were further classified into the following four categories: true positive (actually having ADHD and detected as ADHD, TP in Table 2), false positive (actually normal but detected as ADHD, FP in Table 2), false negative (actually having ADHD but detected as normal, FN in Table 2), and true negative (actually normal and detected as normal, TN in Table 2). From these values, sensitivity was calculated as TP/(TP + FN), where TP + FN is the actual number of ADHD subjects. Specificity was calculated as TN/(TN + FP), where TN + FP is the actual number of normal control subjects.

**Fig. 3:**
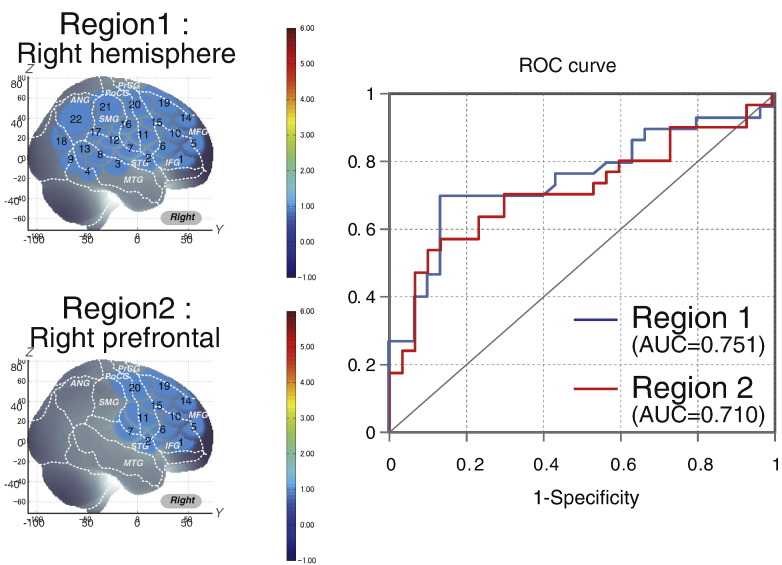
Receiver operating characteristic (ROC) analysis of the oxy-Hb signal contrast in macroanatomical regions of interest (ROIs). Region 1 represents all channels measured in the right hemisphere and region 2 represents the channels located over the right prefrontal cortex.

**Fig. 4:**
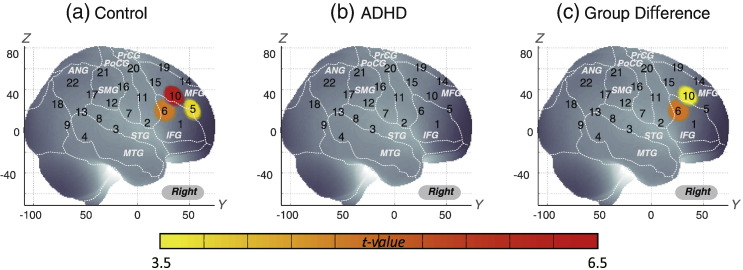
Cortical activation patterns of control subjects (a), ADHD subjects (b) and comparison between the ADHD and control groups (c) during a go/no-go task at the group level. t-maps of oxy-Hb signals are displayed, with significant t-values (paired t-test, Bonferroni-corrected) shown according to the color bar.

**Fig. 5:**
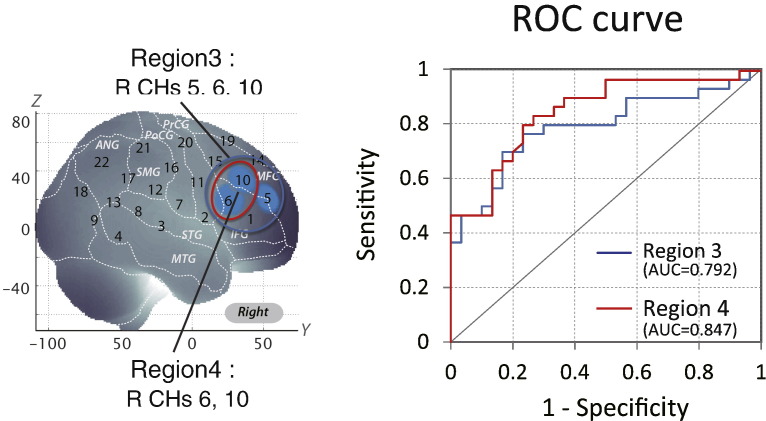
ROC analysis of the averages of oxy-Hb signal contrasts in macroanatomical ROIs for Regions 3 and 4. Region 3 represents the right prefrontal channels activated in typically developing control subjects. Region 4 represents the right prefrontal channels with greater activation in typically developing control subjects than in ADHD children.

**Fig. 6:**
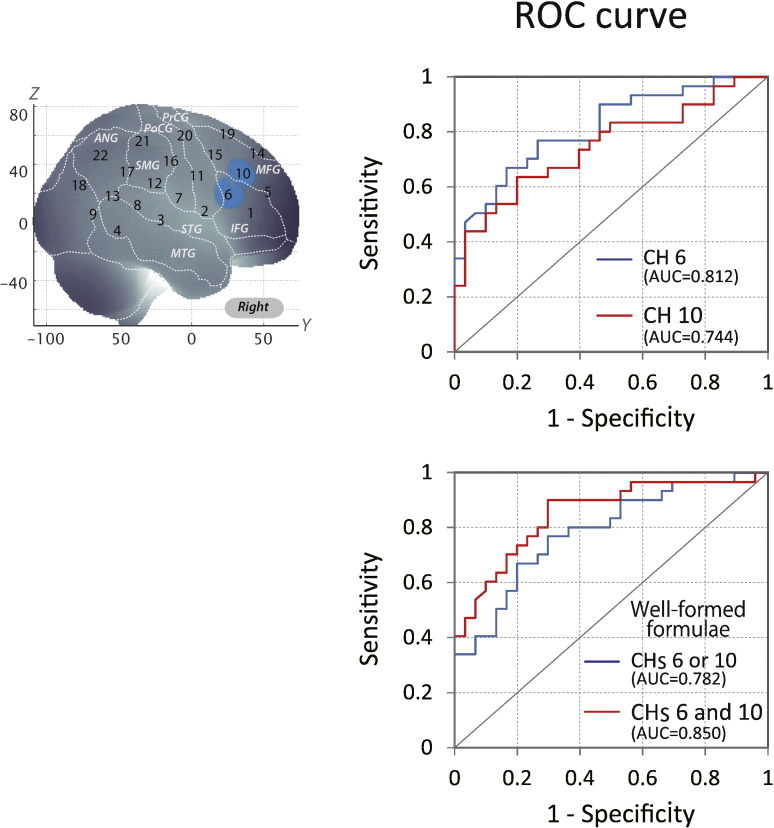
ROC analysis of the average of oxy-Hb signal contrast in the right prefrontal channels optimized using well-formed formulae. The right upper panel represents ROC curves for CHs 6 and 10, respectively. The right lower panel includes the ROC curve adopting OR and AND logics. In OR logic, a patient was identified as normal when either CHs 6 or 10 was activated. In AND logic, a patient was identified as normal when both CHs 6 and 10 were activated.

The initial cut-off value for the oxy-Hb signal was set at 0 mM mm. From this start point, the cut-off value was incremented or diminished until specificity or sensitivity reached 0 or 1. For each cut-off value, sensitivity was plotted against 1-specificity to create an ROC curve. In addition, the area under the resultant ROC curve was calculated. Then we explore the best cut-off value, presenting the best cut-point of sensitivity and specificity, which is the one nearest to the top left corner (Figs. 3, 4, 5, 6).

### 2.6 Modified individual fNIRS-based classification using well-formed formulae

For further optimization, we adapted well-formed formulae for the constituent CHs in the most effective ROI. When AND logic was applied, a subject was classified as normal when his/her oxy-Hb signals for all CHs in the ROI were above a given threshold. When OR logic was applied, a subject was classified as normal when his/her oxy-Hb signal for any one CH in the ROI was above a given threshold. For each classification using well-formed formulae, ROC analysis was performed as described above. We performed all statistical analyses with the PASW statistics (version 18 for Windows) (SPSS Inc., Chicago, USA) software package.

## 3 Results

### 3.1 ROC analysis of the integral value of oxy-Hb signals in region 1 (all channels in the right hemisphere)

Regarding all measured CHs in the right hemisphere (Region 1: CHs 1 to 22), the resulting area under the ROC curve (AUC) value was 75.1%. At the optimal cut-off value of 0.0120 mM mm, differentiation between ADHD and control subjects (Table 3) was achieved with a sensitivity of 70.0% and a specificity of 86.7% (Fig. 3).

### 3.2 ROC analysis of the integral value of oxy-Hb signals in region 2 (right prefrontal cortex)

We calculated the average of the integral value of oxy-Hb in Region 2 (CHs 1, 2, 5, 6, 7, 10, 11, 14, 15, 16, 19 and 20) covering the right prefrontal cortex in 30 individual ADHD and 30 control subjects. Regarding all measured CHs in the right prefrontal cortex, the resulting AUC value was 71.0%. At the optimal cut-off value of 0.0120 mM mm, differentiation between ADHD and control subjects (Table 3) was achieved with a sensitivity of 63.3% and a specificity of 73.3% (Fig. 3).

### 3.3 ROC analysis of the integral value of oxy-Hb signals in region 3 (right prefrontal channels activated in typically developing control subjects)

First, we screened for any fNIRS channels involved in the go/no-go task for control and ADHD subjects at the group level (Fig. 4). We found significant oxy-Hb increase in three CHs in the right (R) hemisphere, including R CH 5 (mean 0.057, SD 0.077, *p* < 0.05, Bonferroni-corrected, Cohen's d = 0.741), R CH 6 (mean 0.046, SD 0.060, *p* < 0.05, Bonferroni-corrected, Cohen's d = 0.755) and R CH 10 (mean 0.068, SD 0.065, *p* < 0.05, Bonferroni-corrected, Cohen's d = 1.046) in control subjects. Conversely, ADHD conditions showed no significant oxy-Hb increase in the measured cortical areas. Thus, we adopted CHs 5, 6 and 10 as statistically specific ROIs to represent the channels activated for go/no-go task execution in typically developing control subjects. We calculated the average of the integral value of oxy-Hb signals for CHs 5, 6 and 10 in 30 individual ADHD and 30 control subjects. The resulting AUC value was 79.2%. At the optimal cut-off value of 0.0240 mM mm; differentiation between ADHD and control subjects (Table 4) was achieved with a sensitivity of 76.7% and a specificity of 76.7% (Fig. 5).

### 3.4 ROC analysis of the integral value of oxy-Hb signals in region 4 (right prefrontal channels with greater activation in typically developing control than in ADHD subjects)

We assessed the group difference in oxy-Hb signals (Fig. 4). The comparison between ADHD and control subjects revealed significant activation of oxy-Hb signal in the right CHs 6 and 10 in the control subjects (independent two-sample t-test; R CHs 6, *p* < 0.05 Bonferroni-corrected, Cohen's d = 0.964; R CHs 10, *p* < 0.05 Bonferroni-corrected, Cohen's d = 0.699). These channels are located in the border region between the right MFG and IFG (R CH 6, MNI coordinates x, y, z (SD): 59,28,19 (25), MFG 18%, IFG 52%; R CH 10, MNI coordinates x, y, z (SD): 48,37,34 (27), MFG 63%, IFG 31%) in reference to a macroanatomical brain atlas (Rorden and Brett, 2000).

Thus, we applied CHs 6 and 10 as statistically specific ROIs to represent greater activation in typically developing control than in ADHD subjects during go/no-go task execution. We calculated the average of the integral value of CHs 6 and 10 for 30 individual ADHD and 30 control subjects. The resulting AUC value was 84.7%. At the optimal cut-off value of 0.0374 mM mm, differentiation between ADHD and control subjects (Table 4) was achieved with a sensitivity of 83.3% and a specificity of 73.3% (Fig. 5).

Furthermore, we examined each channel component specifically. In CH 6, the AUC value was 81.20%. At the optimal cut-off value of 0.0000 mM mm, differentiation between ADHD and control subjects (Table 5) was achieved with a sensitivity of 66.7% and a specificity of 83.3% (Fig. 6). In CH 10, the AUC value was 74.4%. At the optimal cut-off value of 0.0320 mM mm, differentiation between ADHD and control subjects (Table 5) was achieved with a sensitivity of 63.3% and a specificity of 80.0% (Fig. 6).

### 3.5 Modified individual fNIRS-based classification using well-formed formulae

In addition, we adapted well-formed formulae for CHs 6 and 10 to better differentiate ADHD from normal control subjects. When OR logic was adopted, the area under the AUC was 78.2%. At the optimal cut-off value of 0.0650 mM mm, differentiation between ADHD and control subjects (Table 5) was achieved with a sensitivity of 76.7% and a specificity of 70.0% (Fig. 6). Finally, when AND logic was adopted, the AUC value was 85.0%, which was the highest percentage among all classifications. At the optimal cut-off value of 0.0111 mM mm, differentiation between ADHD and control subjects (Table 5) was achieved with a sensitivity of 90.0% and a specificity of 70.0% (Fig. 6).

## 4 Discussion

### 4.1 Overview

In the current study, through individual assessment of channel-wise oxy-Hb signal changes using fNIRS, we successfully identified ROIs in the right IFG and MFG optimal for differentiating ADHD children from typically developing children; adaptation of well-formed formulae to the two CHs to form optimized ROIs achieved a 90% sensitivity for diagnostic predictions in individual subjects. Thus, we suggest the possibility that this novel fNIRS-based method may serve as an efficient diagnostic tool to enable the identification of ADHD children at an individual level.

### 4.2 Right IFG/MFG oxy-Hb signal changes during a go/no-go task as a significant variable for fNIRS-based classification between ADHD and typically developing control children

Initially, we performed an analysis of the whole right hemisphere to achieve a moderately efficient classification method to differentiate between ADHD and typically developing control children resulting in an AUC value of 0.751 and 70% sensitivity. Subsequently, we narrowed the ROIs sequentially to a few CHs in the right IFG/MFG to achieve an AUC value of 0.847 and 83% sensitivity. These results suggest the potential robustness of an fNIRS-based method utilizing oxy-Hb signal changes in the right IFG/MFG during a go/no-go task.

To make further use of oxy-Hb signal data obtained in the right IFG/MFG, we adapted well-formed formulae for two anatomically neighboring CHs. We classified a subject as normal when his/her oxy-Hb signals for all the CHs in the ROI were above a given threshold (AND logic) or when his/her oxy-Hb signals in any CH in the ROI was above a given threshold (OR logic). The use of AND logic led to an improved AUC value of 0.850 and 90% sensitivity. Thus, we suggest that the use of well-formed formulae on macro-anatomically-labeled fNIRS channels is a practical option for augmenting the diagnostic predictivity for ADHD patients at an individual level.

The two CHs used for the optimized prediction of ADHD patients were probabilistically located on the border of the right IFG and MFG. Evidence for abnormal function of the right IFG and MFG in ADHD patients is supported by a wealth of neuroimaging studies. fMRI studies assessing response inhibition have consistently reported right prefrontal activation (Altshuler et al., 2005; Garavan et al., 1999; Mostofsky et al., 2003; Rubia et al., 2001; Simmonds et al., 2008). Moreover, an ALE meta-analysis of go/no-go tasks (Buchsbaum et al., 2005) provided conclusive evidence for the involvement of the right-lateralized network associated with response inhibition, including the right IFG and MFG as reported in (Simmonds et al., 2008). Thus, the use of right IFG and MFG channels for identifying ADHD children is considered relevant from a neuro-functional point of view.

Furthermore, several other fMRI studies have adopted ROC analysis in order to identify the biomarker an individual classification of ADHD children. Recently, an fMRI study performed assessments for the diagnosis of ADHD during a motor-related inhibition task (stop task) (Hart et al., 2014). Hart et al. investigated the neural correlates with inhibition for 30 adolescents with unmedicated and medicated ADHD (ages 10–17) and 30 healthy comparison subjects (ages 10–17). fMRI data showed the pattern of brain activation correctly divided up to 90% of sensitivity and 63% of specificity with an area under the curve value of 81%. The regions of the discriminative network most predictive of controls included right prefrontal area as of our current study. In our current study, we explored the fNIRS-based an individual classification of ADHD children with using ROC analysis. Using the advantages of fNIRS measurement, we investigated the younger ADHD and control subjects including 6 years old because early identification and treatment are important in ADHD children.

In addition, the right IFG and MFG have been reported to innervate the striatum in the dopaminergic (DA) system as well as the locus coeruleus (Singh-Curry and Husain, 2009). Therefore, the hypoactivation in the right IFG and MFG identified using fNIRS with ADHD patients would reflect a monoamine dysfunction of the central nervous system.

### 4.3 Clinical utility

In the present study, we selected a go/no-go task paradigm with alternating go blocks as baseline blocks and go/no-go blocks as target blocks without rest segments in between active (go and go/no-go) task blocks (Tsujii et al., 2011). Tsujii et al. (2011) also adopted a similar block designed for go/no-go tasks, and treated the go task period as the baseline for contrast with the go/no-go task period when analyzing fNIRS signals. This paradigm was set primarily because of the difficulty with ADHD patients staying still while not performing any tasks, which may lead to unexpected movements or hyperactive behavior. In addition, we omitted rest blocks to save time, as a long experiment time would bore ADHD subjects. Furthermore, the go and go/no-go block design is commonly used in fMRI studies (Altshuler et al., 2005; Dillo et al., 2010; Ma et al., 2012; Vaidya et al., 1998). Thus, considering comparisons across modalities, the use of the go/no-go task paradigm in the current study is appropriate.

Another merit of the block-design paradigm is that the baseline blocks serve as a motor control for the target blocks. Schecklmann et al. (2008) used a weekday-reciting task as a baseline block and a word fluency task as a target block, and used fNIRS to analyze the difference in signal between the two tasks. In this paradigm, movement and muscle artifacts during the task condition are expected to be neutralized with the use of a control condition with a similar motor output. Similarly, we adopted the go task as the baseline task. As the physical movements made by children during the go task are similar to those of the go/no-go task, movement and muscle artifacts are expected to be ruled out. Accordingly, activation during the go/no-go task block is considered to reflect inhibitory control; thus, this paradigm is more appropriate than one using a rest block as the baseline. Although fNIRS studies often use a paradigm where rest and task blocks are alternately performed (Herrmann et al., 2005), we suggest that it would be more applicable for studies involving younger ADHD children to adopt the alternating go and go/no-go block design.

Furthermore, reminiscent of our study demonstrating the clinical utility of fNIRS-based method for individual diagnosis of ADHD children offers two significant advantages: it is simple and it is an ADHD-friendly diagnostic tool, applicable to preschool ADHD children as young as 6 years old.

Several previous studies have reported on systems for individual diagnostic classification of ADHD and control subjects that apply multifactorial methods (e.g., neuroanatomical pattern classification) with structural MRI data (Durston et al., 2003; Johnston et al., 2014) and to fMRI data (Colby et al., 2012; Dai et al., 2012; Hart et al., 2014; Liang et al., 2012; Solmaz et al., 2012; Zhu et al., 2008). Conversely, our protocol requires only a single variable (the simple ‘integral value’ for only two regions of fNIRS signals) and produces classification rates (sensitivity: 90%). This is equivalent to those reported in previous MRI and fMRI studies using multivariate statistical methods, which had 67–93% classification rates in ADHD groups compared with typically developing control groups.

In addition, it should be noted that fNIRS-based measurement offers a clinical advantage for the diagnosis of ADHD children: fNIRS is convenient and robust, and thus is suitable for the functional monitoring of children with ADHD, who have difficulty performing active cognitive tasks in the enclosed environments of other imaging modalities. In fact, the cumulative post-scan exclusion rate of our current study was 4.8% (three subjects among 33 ADHD and 30 control children) as in our previous studies which also presented less than 5% of a total of 81 ADHD children and 69 control subject (Monden et al., 2012a; Monden et al., 2012c; Nagashima et al., 2014a; Nagashima et al., 2014b; Nagashima et al., 2014c). This is far lower than the rejection rate of fMRI studies, which is typically 50% for ADHD subjects 6 years old and older and 30% for the corresponding normal controls (Durston et al., 2003). These high rejection rates are mainly due to motion and lack of compliance (Yerys et al., 2009).

fNIRS-based clinical diagnosis of individual subjects is attracting increasing research interest with extremely promising results. A recent multi-site case-control large-scale fNIRS study involving over 600 adult patients with depressive disorder, bipolar disorder, or schizophrenia reported high classification accuracy with univariable analysis, a centroid in the hemodynamic response pattern: sensitivity of discrimination from healthy control subjects was 74.6% for major depressive disorder, 76.9% for bipolar disorder, and 90.0% for schizophrenia (Takizawa et al., 2014). In addition, although sample size was rather small, enrolling nine boys with medicated ADHD and eight boys with autism spectrum disorder (ASD), use of a support vector machine on hemodynamic response data during a task involving viewing the subject's mother's face allowed the distinction of the two populations with an 84% classification accuracy (note this is a different measure from sensitivity) (Ichikawa et al., 2014). These studies imply the feasibility of fNIRS-based single-subject diagnosis with various technical approaches.

### 4.4 Limitations

In the current study, we present a fNIRS-based application and demonstrate its utility for individually classifying ADHD and typically developing control children. However, for appropriate interpretation of the current findings, several issues need to be addressed.

First, the current study aimed at technical exploration with an emphasis on spatial analysis, and thus the sample size was relatively small. In order to validate the clinical utility of the current method, more extensive research with a larger sample size must be performed.

Second, the current study does not necessarily represent an initial screening phase for ADHD diagnosis, but rather corresponds to a reconfirmation process for ADHD-confirmed patients. Therefore, for its potential use as aid for diagnosis, we need to explore disorder-specificity of fNIRS based individual classification relative to other developmental and psychiatric disorders, such as Autistic spectrum disorder, oppositional defiant disorder, conduct disorder, depression, and anxiety in the next step.

Third, although all ADHD subjects had temporally stopped medication (MPH or ATX) more than 48 hours before fNIRS examination, the conditions of the ADHD subjects in this study may not exactly mimic those of naïve ADHD patients. The brain function and structure can be changed by long-term MPH and ATX administration; a recent metaanalysis on human studies using fMRI suggested that long-term MPH treatment is associated with more normal activation in the right DLPFC (Hart et al., 2012). Therefore, the need to examine medication naïve ADHD patients remains, and would be a logical next step.

## 5 Conclusions

The current study intended to examine the practical utility of individual diagnostic classification between ADHD and typically developing children. To our knowledge, this is the first fNIRS-based neuro-diagnostic monitoring method enabling the distinction between ADHD and typically developing children. The fNIRS-based measurements were simple and sufficiently robust, with 90% sensitivity and 70% specificity applicable to all ADHD children (9.2 years of age on average) including those as young as 6 years. The specific prediction of ADHD children was achieved by monitoring hypoactivation of the two channels located at the right IFG and MFG during an inhibitory control task, and was reinforced by the adaptation of well-formed formulae, suggesting the utility of right IFG/MFG hypoactivation as a potential biomarker for individual diagnosis of ADHD patients. The current results clearly demonstrate the potential of fNIRS as a monitoring tool for the early diagnosis of ADHD children, although its actual use in clinical diagnosis of ADHD children should await further validation involving medication naïve ADHD patients.

## Figures and Tables

**Table 1:** Demographic data for control and ADHD subjects.

	Control		ADHD		Control vs. ADHD	
	Mean	SD	Mean	SD	χ^2^*/t*	*p*
Gender (male/female)	25:5		20:10		2.222	0.136^ns^
Age	9.7	2.3	9.1	2.6	0.904	0.370^ns^
WISC-Ⅲ Full IQ	109.3	14.9	95.5	13.0	3.802	0.000**

Abbreviations: WISC-III, Wechsler Intelligence Scale of Children, third edition; IQ, intelligence quotient; SD, standard deviation; χ^2^, Chi-squared; *t*, *t* value; *p*, *p* value. Statistical significances are presented as follows:

** *p* < 0.01; and ns, not significant.

**Table 2:** 

		ADHD	
		Present	Absent
fNIRS-based analysis	Positive result	TP (a)	FP (b)
	Negative result	FN (c)	TN (d)
		Sensitivity a/(a + c)	Specificity d/(b + d)

Abbreviations: TP: true positive (actually having ADHD and detected as ADHD), FP: false positive (actually normal but detected as ADHD), TN: true negative (actually normal and detected as normal), FN: false negative (actually having ADHD but detected as normal).

**Table 3:** Sensitivities and specificities of the integral values of Region 1 and 2 signal changes for each cut-off value.

	Region 1		Region 2	
Cut-off Value (mM mm)	Sensitivity	Specificity	Sensitivity	Specificity
0.0400	86.67	36.67	80.00	40.00
0.0360	83.33	36.67	76.67	40.00
0.0320	80.00	40.00	70.00	46.67
0.0280	80.00	43.33	70.00	53.33
0.0240	76.67	56.67	70.00	63.33
0.0200	70.00	60.00	70.00	66.67
0.0160	70.00	73.33	63.33	70.00
0.0120	70.00	86.67	63.33	73.33
0.0080	60.00	86.67	56.67	80.00
0.0040	60.00	86.67	56.67	86.67
0.0000	56.67	86.67	56.67	86.67
−0.0040	50.00	86.67	53.33	86.67
−0.0080	46.67	86.67	50.00	90.00
−0.0120	46.67	90.00	43.33	93.33
−0.0160	40.00	90.00	40.00	93.33
−0.0200	36.67	93.33	26.67	93.33
−0.0240	30.00	93.33	23.33	93.33
−0.0280	30.00	93.33	23.33	93.33
−0.0320	30.00	93.33	23.33	96.67
−0.0360	26.67	93.33	23.33	96.67
−0.0400	26.67	93.33	20.00	96.67

**Table 4:** Sensitivities and specificities of the integral values of Region 3 and 4 signal changes for each cut-off value.

	Region 3		Region 4	
Cut-off Value (mM mm)	Sensitivity	Specificity	Sensitivity	Specificity
0.0400	80.00	66.67	83.33	66.67
0.0374	80.00	66.67	83.33	73.33
0.0360	80.00	66.67	80.00	76.67
0.0320	80.00	70.00	80.00	76.67
0.0280	76.67	70.00	66.67	80.00
0.0240	76.67	76.67	63.33	83.33
0.0200	70.00	83.33	60.00	86.67
0.0160	60.00	83.33	53.33	86.67
0.0120	56.67	83.33	53.33	86.67
0.0080	56.67	83.33	53.33	86.67
0.0040	56.67	86.67	50.00	86.67
0.0000	56.67	86.67	46.67	86.67
−0.0040	50.00	86.67	46.67	90.00
−0.0080	46.67	93.33	46.67	93.33
−0.0120	46.67	96.67	46.67	100.00
−0.0160	46.67	96.67	46.67	100.00
−0.0200	40.00	96.67	40.00	100.00
−0.0240	36.67	100.00	36.67	100.00
−0.0280	36.67	100.00	33.33	100.00
−0.0320	30.00	100.00	30.00	100.00
−0.0360	26.67	100.00	26.67	100.00
−0.0400	26.67	100.00	26.67	100.00

**Table 5:** Sensitivities and specificities of the integral values of signal changes for CH 6 only, CH 10 only, CH 6 or 10 (well-formed formulae), and CHs 6 and 10 (well-formed formulae) for each cut-off value.

	CH 6		CH 10		CHs 6 or 10 (Well-formed formulae)		CHs 6 and 10 (Well-formed formulae)	
Cut-off Value (mM mm)	Sensitivity	Specificity	Sensitivity	Specificity	Sensitivity	Specificity	Sensitivity	Specificity
0.0650	93.33	43.33	80.00	53.33	76.67	70.00	96.67	26.67
0.0400	83.33	53.33	63.33	73.33	53.33	83.33	93.33	43.33
0.0360	80.00	53.33	63.33	76.67	50.00	86.67	93.33	43.33
0.0320	80.00	53.33	63.33	80.00	50.00	86.67	93.33	46.67
0.0280	76.67	53.33	56.67	80.00	43.33	86.67	90.00	46.67
0.0240	76.67	60.00	53.33	80.00	40.00	86.67	90.00	53.33
0.0200	76.67	66.67	53.33	83.33	40.00	86.67	90.00	63.33
0.0160	76.67	70.00	53.33	86.67	40.00	90.00	90.00	66.67
0.0120	76.67	70.00	53.33	86.67	40.00	90.00	90.00	66.67
0.0111	76.67	73.33	53.33	86.67	40.00	90.00	90.00	70.00
0.0080	70.00	76.67	50.00	86.67	40.00	90.00	80.00	73.33
0.0040	66.67	76.67	50.00	90.00	40.00	93.33	76.67	73.33
0.0000	66.67	83.33	43.33	90.00	33.33	96.67	76.67	76.67
−0.0040	66.67	83.33	43.33	90.00	33.33	96.67	76.67	76.67
−0.0080	63.33	83.33	43.33	93.33	33.33	100.00	73.33	76.67
−0.0120	60.00	83.33	40.00	96.67	30.00	100.00	70.00	80.00
−0.0160	60.00	86.67	40.00	96.67	30.00	100.00	70.00	83.33
−0.0200	53.33	86.67	40.00	96.67	30.00	100.00	63.33	83.33
−0.0240	50.00	93.33	30.00	96.67	20.00	100.00	60.00	90.00
−0.0280	50.00	93.33	26.67	96.67	20.00	100.00	56.67	90.00
−0.0320	40.00	96.67	20.00	100.00	16.67	100.00	43.33	96.67
−0.0360	36.67	96.67	20.00	100.00	16.67	100.00	40.00	96.67
−0.0400	30.00	100.00	20.00	100.00	13.33	100.00	36.67	100.00
